# Hand hygiene knowledge, attitude, barriers and improvement measures among healthcare workers in the Republic of Korea: a cross-sectional survey exploring interprofessional differences

**DOI:** 10.1186/s13756-023-01296-y

**Published:** 2023-09-07

**Authors:** Jaewoong Kim, Shi Nae Yu, Yeon Su Jeong, Jin Hwa Kim, Min Hyok Jeon, Tark Kim, Eun Ju Choo, Eunjung Lee, Tae Hyong Kim, Se Yoon Park

**Affiliations:** 1 Department of Biomedical Systems Informatics, Yonsei University College of Medicine, Seoul, Republic of Korea; 2 Yongin Severance Hospital, Centers for Digital Health, Yonsei University Health System, Yongin, Republic of Korea; 3 Division of Infectious Diseases, Department of Internal Medicine, Soonchunhyang University Cheonan Hospital, Soonchunhyang University College of Medicine, Cheonan, Republic of Korea; 4 Infection Control Team, Soonchunhyang University Seoul Hospital, Seoul, Republic of Korea; 5 Division of Infectious Diseases, Department of Internal Medicine, Soonchunhyang University Bucheon Hospital, Soonchunhyang University College of Medicine, Bucheon, Republic of Korea; 6 Division of Infectious Diseases, Department of Internal Medicine, Soonchunhyang University Seoul Hospital, Soonchunhyang University College of Medicine, Seoul, Republic of Korea; 7 Department of Internal Medicine, Hanyang University College of Medicine, 222 Wangsimni-ro, Seongdong-gu, Seoul, 04763 Republic of Korea

**Keywords:** Hand hygiene, Barriers, Healthcare workers, Occupation, Intervention

## Abstract

**Background:**

Hand hygiene (HH) is a fundamental component of infection prevention and control in healthcare settings. This study aimed to identify knowledge, attitude, and barriers to HH according to occupational groups and strategies to increase the rate of HH compliance among healthcare workers (HCWs).

**Methods:**

This cross-sectional survey was conducted in July 2018 at four university-affiliated hospitals. The survey comprised seven parts with 49 items, including self-reported HH compliance, knowledge, attitudes, behaviours, barriers to HH, and improvement strategies.

**Results:**

A total of 1046 HCWs participated in the survey. The nursing group’s self-reported HH compliance rate was the highest, followed by other HCWs and physicians. The scores regarding knowledge, attitudes, and behaviours regarding HH were the highest in the nursing group. The nursing group also had higher internal and emotional motivation scores. Physicians and nurses found HH the most challenging in emergencies, while other HCWs considered skin problems caused by HH products the most significant barrier. Among 12 improvement measures, approximately 20% of the respondents ranked “diversify types of hand sanitisers,“ “install soap and paper towels in each hospital room,“ and “change perception through various HH campaigns” as the top three priorities. The physician group deemed the timely reminder of HH compliance as the second most critical improvement measure.

**Conclusion:**

Differences in knowledge, attitude and barriers hindering HH compliance and improvement plans were identified for each group. The findings suggest that targeted interventions tailored to the specific needs of different occupational groups may effectively improve HH compliance in healthcare settings.

**Supplementary Information:**

The online version contains supplementary material available at 10.1186/s13756-023-01296-y.

## Background

Healthcare-associated infections (HAIs) are estimated to occur in 7–10% of hospitalised patients globally and cause socioeconomic losses, including longer hospital stays, increased healthcare costs, lower quality of care, and healthcare disputes [1, 2]. According to the World Health Organization (WHO), hand hygiene (HH) is considered the most important activity for preventing HAIs [3]. Since the WHO released guidelines for implementing HH promotion programs in 2009, the Republic of Korea (ROK) implemented a pilot project for a national HH campaign in 2013. The campaign aims to establish a consistent and uniform program for improving HH, using the WHO multimodal HH improvement strategy. As a result, there has been an improvement in HH promotion activities in Korean hospitals [4]. Despite these efforts, reported rates of HH compliance among healthcare workers (HCWs) in Korean hospitals remain suboptimal, ranging from 30–60% [5–7]. Various strategies to improve HH performance can be effective in the short and long term [8, 9].

Previous studies on barriers and facilitators to HH have suggested a lack of role models, including coworkers and supervisors, skin irritation from hand sanitisers, lack of manpower or time, scepticism about the effectiveness of HH, and a lack of an institutional culture that values HH [10, 11]. In addition, it is reported that physicians’ HH performance is lower than nurses’ [5, 12, 13]. Qualitative and small-scale quantitative studies on strategies to overcome these barriers reveal that HH compliance rates can be increased through a multifaceted approach such as educational activities, monitoring and feedback, and placement of HH tools [14, 15]. However, there is still a lack of large-scale quantitative studies on barriers to HH compliance and studies considering different occupational groups of HCWs.

Therefore, this study aimed to investigate HH knowledge, attitudes, importance, and achievement, as well as barriers and improvement measures, to investigate differences among occupations and establish improvement strategies for each.

## Methods

### Study design

This cross-sectional survey was conducted at four university-affiliated hospitals in the Republic of Korea. The four hospitals that participated in the study are located in different regions and are labelled Hospitals A, B, C, and D. The study participants were all HCWs in the study hospitals. At the time of the survey, 6048 HCWs were identified as potential participants. The questionnaires were distributed and collected through the infection control team of each hospital. The data collection period was from July 9 to July 22, 2018.

### Survey items

Survey items were selected and modified from a previous study conducted by Ibrahim et al. [16].

The survey included a structured questionnaire with seven parts: (a) self-assessment of HH and optimal HH compliance rate, knowledge, attitudes, and behaviours regarding HH (11 questions), (b) internal motivation for better HH (8 questions), (c) obstacles for HH (14 questions), (d) emotional motivation (3 questions), (e) need for external supervision (4 questions), (f) preference for alcohol gel (3 questions), and (g) embarrassment due to supervision (2 questions).

The HH compliance rate was self-assessed and determined by dividing the number of observed HH actions by the number of opportunities. The opportunities for HH consisted of the WHO’s “5 opportunities for hand hygiene.” Optimal HH compliance rates were determined based on self-assessed adherence to the WHO-recommended six-step technique with appropriate time at each opportunity [17].

Ten HH promotion activity items divided into importance and achievement categories were selected and adapted from a previous study [18]. The items include hand sanitiser placed where necessary, regular hand hygiene education, practical training according to the situation, frequent monitoring, department-wide feedback, personal feedback, hand hygiene information poster, audiovisual alarming/guidance, management’s interest and encouragement, and reward and publicise excellent hand hygiene employees/departments. Each item consisted of a five-point Likert scale, and data were collected by evaluating the importance and achievement of the same item. In addition, we collected information on the frequency of education within each department provided by the infection control team in person or online using a five-point Likert scale. Other items, such as internal, emotional motivation, and barriers to HH, consisted of 14 items on a seven-point Likert scale. A higher score meant higher agreement. The method used to improve HH involved participants selecting three methods from a pool of 12 options and then indicating the preferred order for these chosen methods.

### Statistical analysis

Statistical analysis was performed using R software (version 4.2.2; https://www.r-project.org/). We compared HH compliance among occupations using a t-test and analysis of variance (ANOVA). A post-hoc analysis was performed, and Scheffe’s method was used. A scatter plot was used to compare the relationship between importance and achievement collected on a five-point Likert scale. A *P*-value of < 0.05 was considered statistically significant. Cronbach’s alpha coefficients were obtained to verify the internal consistency of the measures. A reliability coefficient of 0.7 or higher is considered acceptable, and 0.8 or higher is considered highly reliable.

### Ethics statement

The study protocol was approved by the institutional review board (IRB) of Soonchunhyang University Seoul Hospital. The requirement for written informed consent from the participants was waived for the analysis of the conducted survey results.

## Results

This study included 1046 participants: 734 (70.2%) nurses (nurses and nursing assistants), 203 (19.4%) physicians (interns, residents, and specialists), and 109 (10.4%) other HCWs (medical engineers, transport agents, physiotherapists, pharmacists, clinical pathologists, and hospital custodians). The response rate among different ranks of HCWs was highest in nurses with 23.9% (734/3,065), followed by physicians with 17.4% (203/1,167) and other HCWs with 6.0% (109/1,806). The response rate of each hospital (A, B, C, and D) was 21.9%, 12.5%, 16.7%, and 22.6%, respectively. Detailed demographics are shown in Table 1. The Cronbach’s alpha coefficients were 0.950 in the survey items for ‘knowledge, attitudes, and behaviour regarding HH’, 0.878 for ‘internal motivation for better HH’ and 0.926 for ‘obstacles to HH’.

**Table 1:** Demographic information of the study participants

Characteristics	Categories	Total (N = 1046)
Occupational categories	Nurse (nurse and nursing assistant)	734 (70.2)
	Physician (intern, resident, and specialists)	203 (19.4)
	Other healthcare workers (medical engineers, transport agents, and others)	109 (10.4)
Working area	Inpatient wards	363 (49.9)
	Outpatient clinic	143 (19.6)
	Emergency department	32 (4.4)
	Intensive care unit	81 (11.1)
	Others	109 (15.0)
Gender	Female	855 (81.9)
Age groups	20–29	410 (39.4)
	30–39	306 (29.4)
	40–49	245 (23.6)
	50–59	79 (7.6)
Working experience	< 6 months	80 (9.5)
	≤ 6 months to < 1 year	42 (5.0)
	≤ 1 year to < 3 years	119 (14.1)
	≤ 3 years to < 5 years	104 (12.3)
	≤ 5 years to < 10 years	148 (17.6)
	≤ 10 years to < 20 years	188 (22.3)
	≥ 20 years	162 (19.2)
Allergy to alcohol hand sanitiser	Yes	117 (11.3)

Data are expressed as numbers (%) unless otherwise specified

### Self-reported hand hygiene compliance rate

**Fig. 1:**
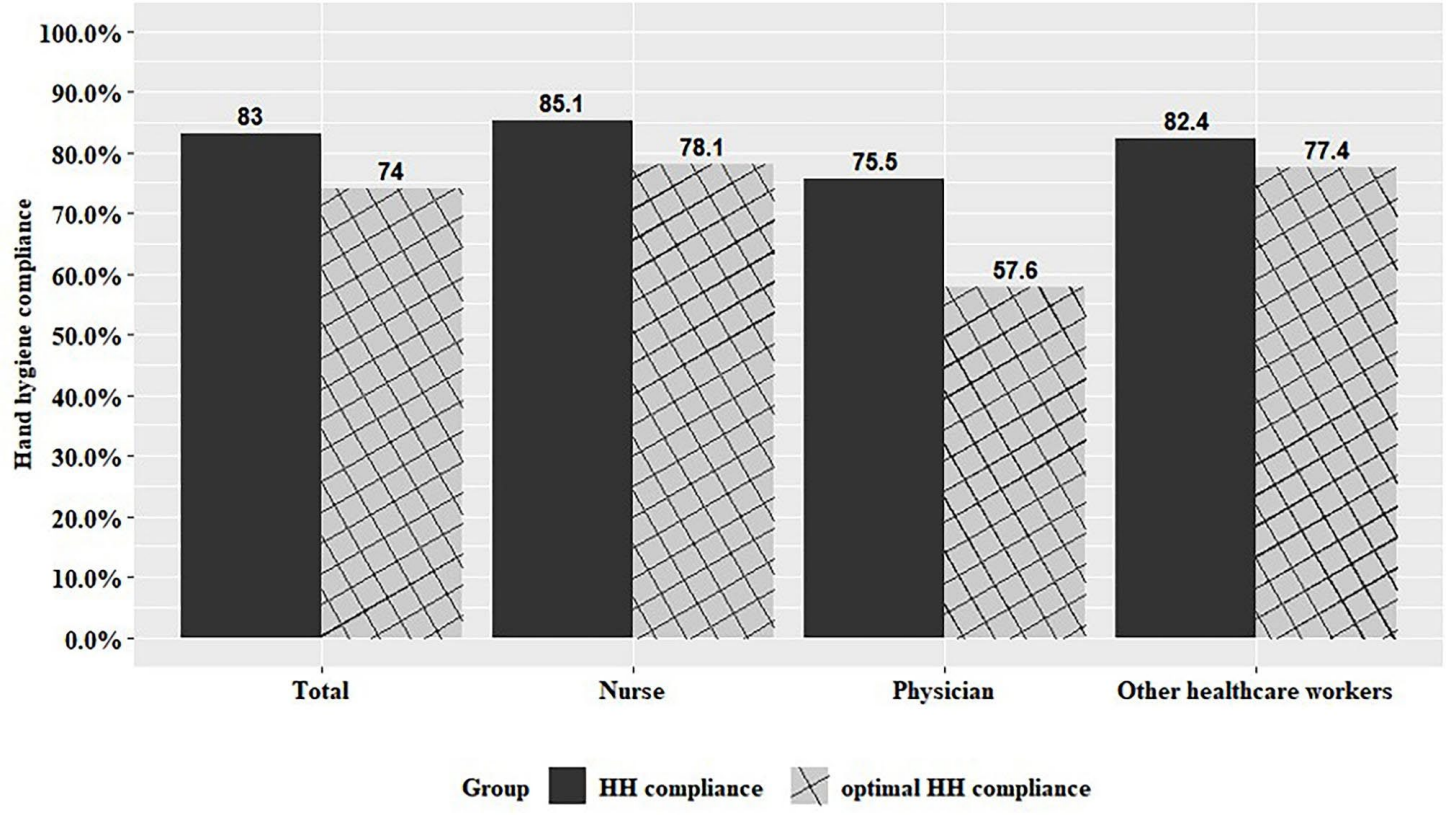
Hand hygiene and optimal hand hygiene compliance rate

The mean self-reported HH and optimal HH compliance rates were 84.0% and 74.0%, respectively. The HH and optimal HH compliance were highest in the nursing group, followed by other HCWs and physicians. The HH and optimal HH compliance among occupational groups were significantly different, except at Hospital D. However, at Hospital B, other HCWs showed higher HH and optimal HH compliance than nurses, and at Hospital C, other HCWs showed higher optimal HH compliance than nurses, but this was not statistically significant (Fig. 1, Supplemental Table A1). The post hoc analysis revealed the difference in HH and optimal HH compliance between physicians and the other groups (nurses and HCWs). Among five opportunities for HH, the physician group had the lowest compliance before or after touching a patient and after touching the patient’s surroundings (Supplemental Figure A1).

### Relationship between importance and achievement

**Fig. 2:**
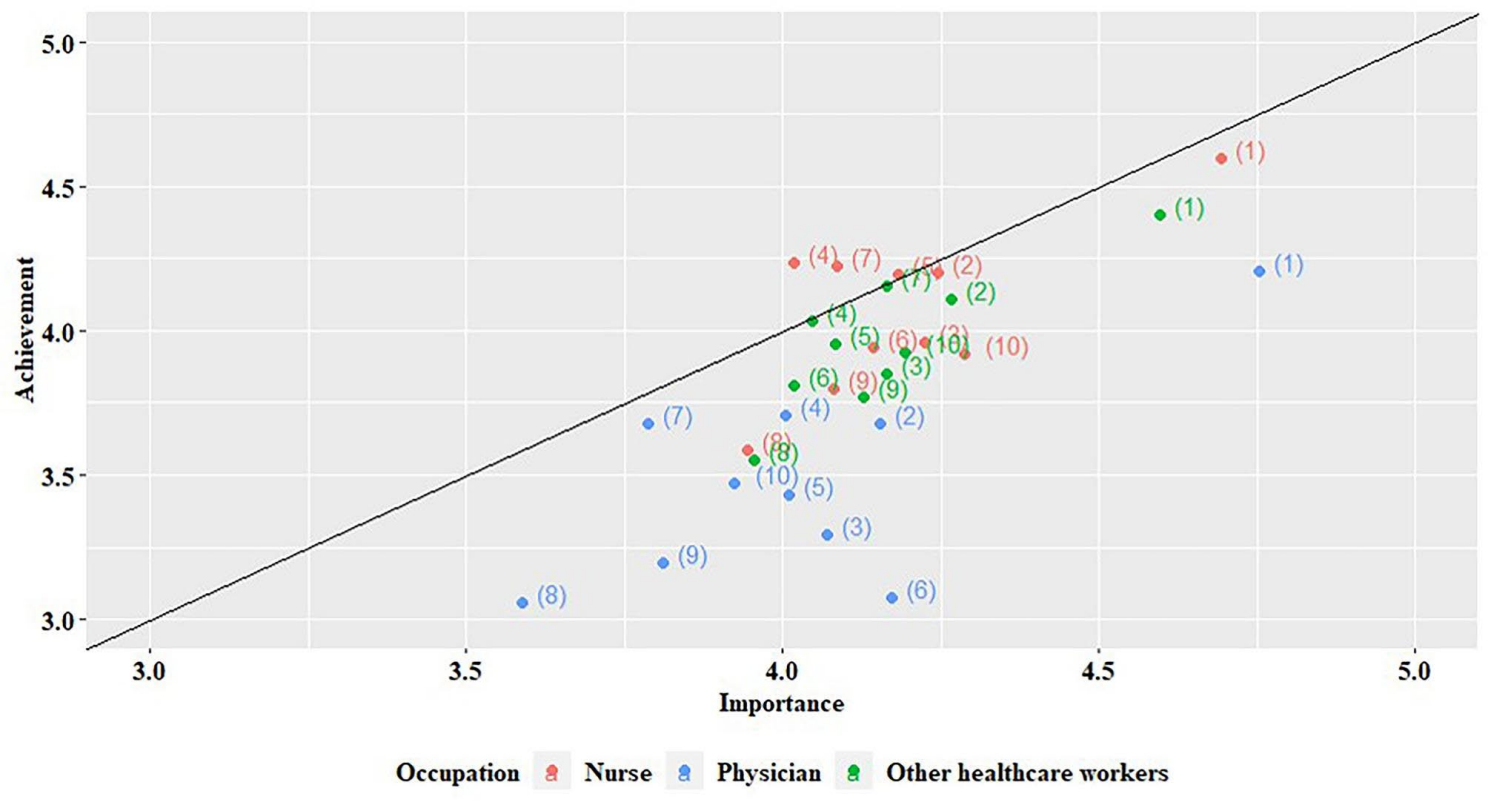
Relationship between importance and achievement scores for hand hygiene improvement measures. The graph shows the importance and achievement scores for each measure on a scale of 1–5, with 1 being low and 5 being high. The measures include hand sanitiser placed where necessary (1), regular hand hygiene education (2), practical training according to the situation (3), frequent monitoring (4), department-wide feedback (5), personal feedback (6), hand hygiene information poster (7), audiovisual alarming/guidance (8), management’s interest and encouragement (9), and reward and publicise excellent hand hygiene employees/departments (10)

The relationship between importance and achievement is shown in Fig. 2. The solid line in the centre is where the importance and achievement scores are the same. A score above the line means more achievement than importance, and below the line means less achievement than importance. There was a significant correlation between importance and achievements (*P* value < 0.01, Supplemental Table A2). The most important intervention was “hand sanitiser placed where necessary,” followed by “regular HH education” and “reward and publicise excellent HH employees/departments.” The distribution of the nurses, physicians, and other HCWs was similar, and a downward bias was observed for the physicians. Only three interventions for nurses (“frequent monitoring,” “department-wide feedback,” and “HH information poster”) were associated with higher achievement than importance. In the physicians group, “HH information poster,” “audiovisual alarming/guidance,” “management’s interest and encouragement,” and “reward and publicise excellent HH employees/departments” scored significantly lower in importance than those in the nurses or other HCW group. Furthermore, achievement was significantly lower for physicians than nurses or other HCWs in the post-hoc analysis (Supplemental Table A3).

### Hand hygiene education

Across all occupations, HH education was most often provided once or twice a year, followed by once or twice a quarter. However, HH education was never provided in the physicians group, which was higher than in other occupational groups. Additionally, education provided more than once or twice a year was the lowest in the physicians group (Supplemental Figure A2).

### Knowledge, attitudes, and behaviours regarding hand hygiene

The scores regarding knowledge, attitudes, and behaviours about HH were highest in the nurses group. Except for the item “Jewelry and artificial nails make your hands more germ-friendly,” there were significantly different scores between occupations. In the post hoc analysis, the physicians group showed the lowest score for the following five questions, “I do hand hygiene before contacting the patient.”, “It is easy to cleanse hands because alcohol gel is close.”, “If I do not do hand hygiene, I can get infected too.”, “My patient expects me to do my HH well” and “The performance of HH by colleagues affects my HH rate” (Table 2, Supplemental Table A4).

**Table 2:** Knowledge, attitudes, and behaviours about hand hygiene among healthcare workers

Variables	Total		Nurse		Physician		Other healthcare workers		*P* value
	Mean	SD	Mean	SD	Mean	SD	Mean	SD	
Hand hygiene is important to maintaining my professionalism.	6.35	1.014	6.42^a^	0.982	6.29	0.917	6.06^b^	1.304	< 0.001
I know when to do hand hygiene.	6.50	0.868	6.57^a^	0.852	6.34^b^	0.709	6.27^b^	1.136	< 0.001
I know the correct hand hygiene method (action).	6.51	0.870	6.59^a^	0.848	6.33^b^	0.748	6.3^b^	1.118	< 0.001
I do hand hygiene before contacting the patient.	6.14	1.064	6.26^a^	0.990	5.81^b^	1.129	5.91^b^	1.244	< 0.001
Hand hygiene is a part of medical practice.	6.44	0.925	6.49	0.883	6.34	0.896	6.28	1.193	0.022
It is easy to cleanse hands because alcohol gel is close.	6.38	1.012	6.51^a^	0.894	6.06^b^	1.077	6.12^b^	1.393	< 0.001
If I do not do hand hygiene, I can get infected too.	6.51	0.912	6.56^a^	0.865	6.36^b^	0.983	6.42	1.048	0.012
My patient expects me to do my hand hygiene well.	6.28	1.011	6.33^a^	0.996	6.13^b^	0.964	6.21	1.163	0.028
I believe that hand hygiene blocks the spread of infection.	6.42	0.924	6.46	0.904	6.31	0.898	6.29	1.074	0.045
The performance of hand hygiene by colleagues affects my performance.	6.05	1.222	6.16^a^	1.207	5.7^b^	1.208	5.96	1.224	< 0.001
Jewellery and artificial nails make your hands more germ-friendly.	6.34	1.043	6.38	1.067	6.32	0.864	6.16	1.164	0.112

SD, standard deviation

^a,b^ indicate a significant mean difference between groups a and b

### Internal and emotional motivation

Nurses scored higher on all internal and emotional motivation variables, indicating more positive motivation toward HH than physicians and other HCWs. For most variables, ANOVA tests indicated significant differences between the groups, with nurses having higher means than physicians and other HCWs (Table 3, Supplemental Table A5).

**Table 3:** Internal and emotional motivation regarding hand hygiene among healthcare workers

Variables	Total		Nurse		Physician		Other healthcare workers		*P* value
	Mean	SD	Mean	SD	Mean	SD	Mean	SD	
I do hand hygiene to become a role model to my colleagues.	4.66	1.71	4.82^a^	1.69	4.32^b^	1.68	4.20^b^	1.76	< 0.001
Hand hygiene posters and screensavers help with hand hygiene.	5.06	1.46	5.17^a^	1.46	4.59^b^	1.44	5.21^a^	1.39	< 0.001
I want to receive feedback on hand hygiene and improve my performance.	5.13	1.42	5.18	1.45	5.01	1.24	5.00	1.47	0.195
I can do better hand hygiene if the sink is near.	5.61	1.38	5.71^a^	1.38	5.25^b^	1.32	5.63	1.46	< 0.001
Soap or hand towels are provided in each hospital room for good hand hygiene.	5.41	1.70	5.49^a^	1.74	5.05^b^	1.56	5.59^a^	1.56	0.003
If I could get a promotion for hand hygiene, I would do better hand hygiene.	5.26	1.64	5.40^a^	1.61	4.92^b^	1.65	4.94^b^	1.69	< 0.001
Our hospital staff regularly receive feedback on hand hygiene practices.	5.62	1.29	5.83^a^	1.19	4.90^b^	1.39	5.57^a^	1.24	< 0.001
Hand hygiene is the most important in my work.	5.48	1.39	5.70^a^	1.25	4.63^b^	1.57	5.53^a^	1.34	< 0.001

SD, standard deviation

^a,b^ indicate a significant mean difference between groups a and b

### Barriers to hand hygiene compliance

Table 4 and Supplemental Table A6 show the barriers to HH compliance. Among 14 barriers, the top five were “HH is difficult in an emergency,” “HH makes your hands painful and dry,” “It is hard to tell my colleagues to do HH,” “HH wastes time for more important things,” and “It is difficult to do HH if a superior does not do HH.” The top barriers for each occupation were “HH is difficult in an emergency” in the nurses and physicians groups and “HH makes your hands painful and dry (skin trouble)” in the other HCW group.

**Table 4:** The barriers to hand hygiene compliance

Variables	Total		Nurse		Physician		Other healthcare workers		*P* value
	Mean	SD	Mean	SD	Mean	SD	Mean	SD	
Hand hygiene makes your hands painful and dry (skin trouble).	4.47	1.86	4.66^a^	1.82	3.88^b^	1.84	4.32^b^	1.92	< 0.001
It is difficult to do hand hygiene if a superior does not do hand hygiene.	3.04	1.81	2.98	1.82	3.31	1.77	2.94	1.82	0.059
Hand hygiene is difficult in an emergency.	4.99	1.68	5.17^a^	1.66	4.76^b^	1.62	4.22^c^	1.70	< 0.001
It is hard to tell my colleagues to do hand hygiene.	3.53	1.77	3.52	1.82	3.67	1.59	3.31	1.72	0.241
Hand hygiene wastes time for more important things.	3.26	1.74	3.42^a^	1.80	2.98^b^	1.53	2.72^b^	1.51	< 0.001
Hand hygiene is not necessary if you wear gloves.	2.32	1.64	2.21^b^	1.64	2.75^a^	1.59	2.21^b^	1.62	< 0.001
I do not think there is any ethical problem even if I don’t sterilise my hands.	2.27	1.57	2.26	1.59	2.33	1.54	2.21	1.52	0.788
Hand hygiene has not become a habit.	2.64	1.67	2.53^b^	1.65	3.01^a^	1.64	2.72^a^	1.78	0.001
I often forget about hand hygiene.	2.85	1.68	2.73^b^	1.67	3.34^a^	1.63	2.74^b^	1.68	< 0.001
There is no special disadvantage even if hand hygiene is not done.	2.39	1.66	2.28^b^	1.65	2.82^a^	1.67	2.28^b^	1.56	< 0.001
I do not know exactly when to do hand hygiene.	2.00	1.52	1.93^b^	1.54	2.32^a^	1.45	1.94	1.45	0.005
If hand hygiene is being monitored, I do not want to do it.	3.01	1.96	3.06	2.03	3.00	1.79	2.72	1.76	0.246
I am not sure if hand hygiene is helpful for patient safety.	2.01	1.54	2.00	1.58	2.10	1.47	1.92	1.42	0.562
Because there is no soap or hand towel in each hospital room, proper hand hygiene is difficult.	2.66	1.82	2.56^b^	1.87	3.11^a^	1.62	2.50^b^	1.72	< 0.001

SD, standard deviation

^a,b,c^ indicate a significant mean difference between groups a, b and c

### Improvement measures for barriers to performing hand hygiene

**Fig. 3:**
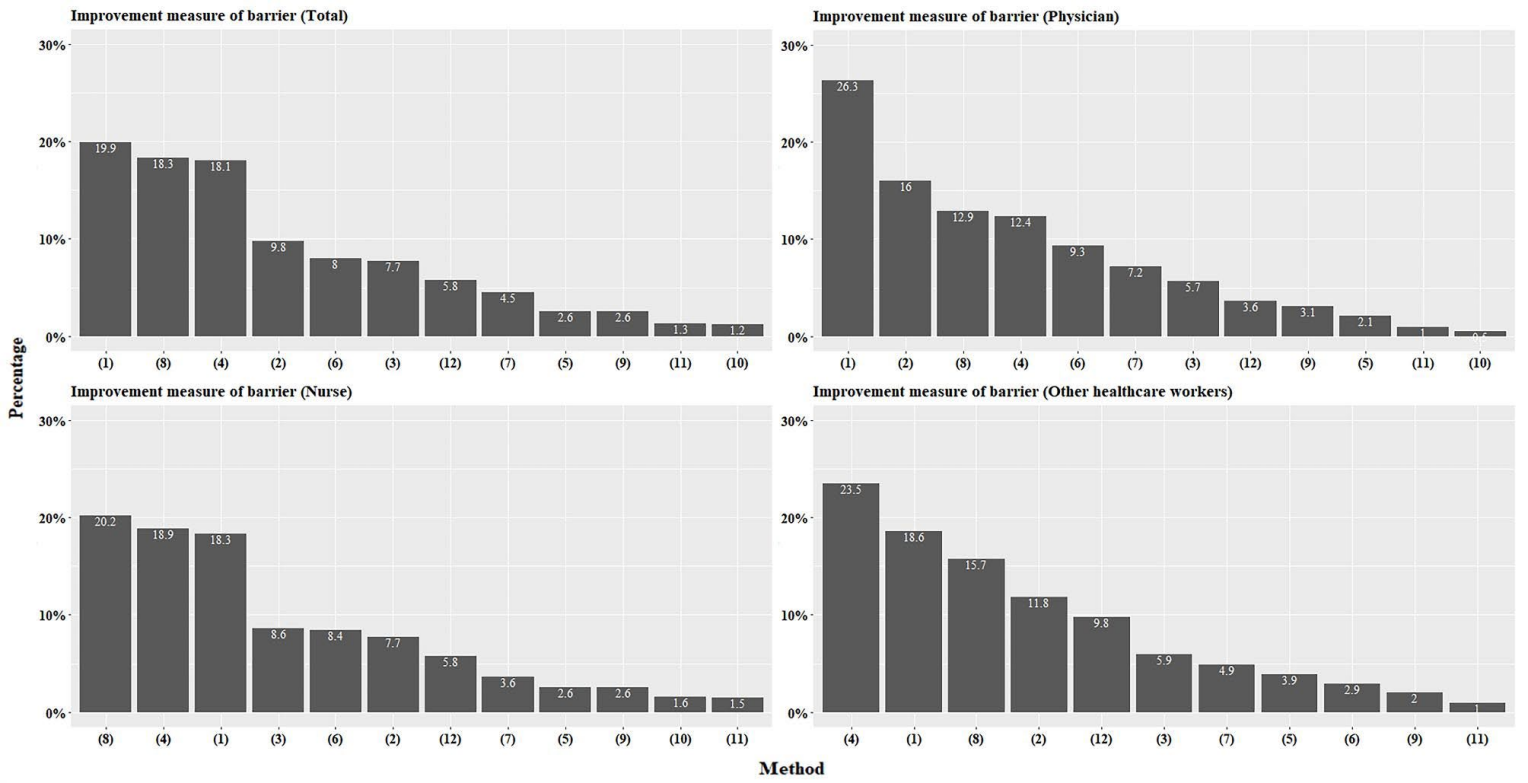
Improvement measures for barriers to performing hand hygiene based on first choice by respondents. The graph shows the percentage of respondents who selected each improvement measure as their first choice. The measures include offering different types of hand sanitisers (1), sending reminders about hand hygiene timing (2), educating patients and caregivers to promote a culture of hand hygiene among staff (3), using hand hygiene campaigns to change perceptions (4), including hand hygiene results in staff performance reviews (5), providing immediate feedback on hand hygiene observations (6), conducting regular monitoring of hand hygiene practices (7), ensuring soap and paper towels are available in all hospital rooms (8), implementing a real-name system to track hand hygiene performance (9), conducting peer-to-peer assessments of hand hygiene performance (10), strengthening hand hygiene theory education (11), and providing training for different hand hygiene situations (12)

Among 12 improvement measures, “diversify types of hand sanitisers,” “install soap and paper towels in each hospital room,” and “change perception through various HH campaigns” were the top three important priorities for 20% of respondents (Fig. 3). The top improvement measures varied among groups. For the nurses group, it was “Install soap and paper towels in each hospital room”; for the physicians group, it was “Diversify types of hand sanitisers”; and for the other HCW group, it was “Change perception through various HH campaigns.” For the physicians group, “Timely reminder of HH compliance” was the second improvement measure (Fig. 3, Supplemental Table A7). The top three important priorities were the same in all four hospitals (Supplemental Fig. 3). When grouped by age, the 40 years or more age group responded, “Change perception through various hand hygiene campaigns” was the most important (Supplemental Tables A8, 9, 10, and 11).

The need for an external reminder had the highest score in “influenced by hospital leaders” (mean 4.95), followed by “reminder by colleagues” (4.73) and “monitoring” (4.49). The physicians group frequently forgot about HH and needed more “monitoring” than other groups (Supplemental Tables A12 and 13).

## Discussion

This study identified differences and similarities in knowledge, attitudes, motivations, and barriers to and strategies for improving HH practices among different healthcare occupations. In particular, the study results provided insights for enhancing HH practices, especially in physicians. Self-reported HH adherence was highest among nurses, followed by other HCWs, and lowest among physicians for HH and optimal HH compliance. Notably, compared to other occupations, physicians had lower HH adherence before and after patient contact and after contact with the environment. Furthermore, physicians scored lower than nurses on knowledge, attitudes, behaviour assessment items, and internal and emotional motivation. Our findings indicate that physicians are less likely to receive HH training compared to nurses and other HCWs. This pattern holds true regardless of the delivery method, whether within departments, through the infection control team, or online. As a result, it may be necessary to implement strategies to actively involve physicians in HH education, such as providing education during office visits.

The achievement of HH promotion activities fell short of their importance across all job categories, indicating the need for more institutional intervention. The highest priority was given to improving the accessibility of hand sanitisers for all job categories. Although HH gel was available in every patient room during the survey, greater accessibility was deemed necessary. However, it should be noted that this survey was conducted before the coronavirus disease 2019 (COVID-19) pandemic and results might have been different if the survey had been conducted after the pandemic. There was the largest gap between the importance and achievement of HH promotion activities among physicians in personal feedback. Providing individual feedback to physicians should be emphasised to address this gap. Additionally, it is important to develop strategies that increase participation in HH promotion activities among physicians by applying these activities within the hospital.

Interestingly, similar response rankings for barriers to and solutions for HH compliance were observed across all four hospitals. The highest-scored barrier to HH compliance was the difficulty performing HH during emergencies. Several studies have shown that HH adherence decreases as workload increases [19, 20]. In particular, HH performance decreased dramatically when there were more than 30 opportunities to perform HH per hour. Strategies that focus more on improving HH performance before critical procedures are needed, as suggested by Chang et al. [20]. Furthermore, devising strategies that can reduce both time and steps while maintaining efficacy is a challenging aspect. A study reported that simplifying HH using the WHO’s six-step technique showed equivalent effectiveness in HH compliance [21]. However, a systematic review reported that the six-step technique was more effective in reducing microbial loads on HCWs’ hands, highlighting the need for well-designed additional studies [22]. The second highest barrier was skin trouble, which was also the highest-ranked solution across all four hospitals, indicating a shared concern that should be addressed collaboratively by all healthcare institutions.

In particular, forgetting about HH was significantly higher among the physicians group, who also ranked “reminder” as the second highest improvement measure. The need for monitoring and personal feedback was also emphasised, with higher responses than other professions. Utilising posters as a reminder may be a natural strategy to improve HH. In one study, posters during ward rounds improved HH among physicians [23]. Additionally, previous evidence indicates that providing feedback on HH performance according to medical speciality improved HH compliance [24]. Moreover, regardless of profession, respondents recognised the importance of leadership in improving HH compliance. A preliminary study also confirmed that leadership affects HH compliance among followers [11]. In addition, the role of peer reminders was identified, and this strategy could be utilised to improve HH [25].

There are some limitations to our study. First, the HH compliance rate was evaluated using self-reported data, which may not accurately reflect the actual HH compliance rate. Previous studies showed that self-reported compliance tends to be generally higher than observed compliance. Therefore, it is crucial to interpret the findings with this potential bias in mind [26, 27]. Second, the study was conducted before the COVID-19 pandemic and, therefore, did not account for any changes in awareness, attitudes toward HH, or accessibility of HH gels that may have occurred since then.

## Conclusions

This study investigated differences in HH practices among various occupations and proposed strategies for improving them. Specifically, for physicians, individual feedback and promotion of participation in related activities were found to be necessary. The difficulty of performing HH during emergencies was a common issue across all occupations. Furthermore, there is a need to improve accessibility and diversity in using HH gels.

### Electronic supplementary material

Below is the link to the electronic supplementary material.

Supplemental Fig. 1. Hand hygiene and optimal hand hygiene compliance rate for each hospital.

Supplemental Fig. 2. Comparison of education frequency among healthcare workers.

Supplemental Fig. 3. Improvement measures for barriers to performing hand hygiene based on first choice by respondents. The graph shows the percentage of respondents who selected each improvement measure as their first choice. The measures include offering different types of hand sanitisers (1), sending reminders about hand hygiene timing (2), educating patients and caregivers to promote a culture of hand hygiene among staff (3), using hand hygiene campaigns to change perceptions (4), including hand hygiene results in staff performance reviews (5), providing immediate feedback on hand hygiene observations (6), conducting regular monitoring of hand hygiene practices (7), ensuring soap and paper towels are available in all hospital rooms (8), implementing a real-name system to track hand hygiene performance (9), conducting peer-to-peer assessments of hand hygiene performance (10), strengthening hand hygiene theory education (11), and providing training for different hand hygiene situations (12)

Supplementary Material 4

## Data Availability

The datasets analysed during the current study are available from the corresponding author upon reasonable request.

## Declarations

### Abbreviations

ANOVA, analysis of variance.

COVID-19, coronavirus disease 2019.

HAIs, healthcare-associated infections.

HCWs, healthcare workers.

HH, hand hygiene.

IRB, institutional review board.

WHO, World Health Organization.
