# Supplementary material for: Hand hygiene knowledge, attitude, barriers and improvement measures among healthcare workers in the Republic of Korea: a cross-sectional survey exploring interprofessional differences
Source: Antimicrob Resist Infect Control. 2023 Sep 7;12:93. doi: 10.1186/s13756-023-01296-y (PMC10483734; doi:10.1186/s13756-023-01296-y)
Supplement: Supplementary file 1 — Supplemental Fig. 1. Hand hygiene and optimal hand hygiene compliance rate for each hospital. [file 13756_2023_1296_MOESM1_ESM.docx]

**Supplemental Table A1.** Hand hygiene and optimal hand hygiene compliance rate

| Hospital | Variables | Total | | | Nurse | | | Physician | | | Other healthcare workers | | | *P* value |
| --- | --- | --- | --- | --- | --- | --- | --- | --- | --- | --- | --- | --- | --- | --- |
|  |  | N | Mean | SD | N | Mean | SD | N | Mean | SD | N | Mean | SD |  |
| Total | HH compliance | 1,046 | 83.0 | 15.1 | 734 | 85.1^a^ | 12.9 | 203 | 75.5^b^ | 19.8 | 109 | 82.4^a^ | 14.3 | <0.001 |
|  | Optimal HH compliance |  | 74.0 | 22.8 |  | 78.1^a^ | 19.3 |  | 57.6^b^ | 28.1 |  | 77.4^a^ | 19.3 | <0.001 |
| Hospital A | HH compliance | 353 | 83.8 | 13.0 | 247 | 86.3^a^ | 10.4 | 57 | 74.9^c^ | 17.8 | 49 | 81.0^b^ | 13.8 | <0.001 |
|  | Optimal HH compliance |  | 74.5 | 20.8 |  | 79.9^a^ | 16.2 |  | 52.3^b^ | 24.8 |  | 73.5^a^ | 19.5 | <0.001 |
| Hospital B | HH compliance | 240 | 78.4 | 14.9 | 144 | 79.0^a^ | 12.7 | 67 | 75.3^a^ | 18.5 | 29 | 82.8^a^ | 14.8 | 0.056 |
|  | Optimal HH compliance |  | 67.7 | 23.4 |  | 69.3^a^ | 20.3 |  | 59.0^b^ | 28.3 |  | 80.8^a^ | 18.0 | <0.001 |
| Hospital C | HH compliance | 317 | 83.6 | 17.0 | 243 | 86.0^a^ | 14.6 | 52 | 72.0^b^ | 22.8 | 22 | 83.6^a^ | 15.4 | <0.001 |
|  | Optimal HH compliance |  | 75.2 | 24.5 |  | 79.9^a^ | 20.4 |  | 51.4^b^ | 29.6 |  | 80.3^a^ | 19.0 | <0.001 |
| Hospital D | HH compliance | 136 | 87.6 | 13.6 | 100 | 88.6^a^ | 11.8 | 27 | 84.5^a^ | 18.7 | 9 | 85.6^a^ | 14.2 | 0.356 |
|  | Optimal HH compliance |  | 80.8 | 19.9 |  | 81.7^a^ | 18.9 |  | 77.4^a^ | 22.8 |  | 80.6^a^ | 22.4 | 0.614 |

SD, standard deviation; HH, hand hygiene.

^a, b^ indicate a significant mean difference between groups a and b.

**Supplemental Table A2.** Relationship between importance and achievements in hand hygiene promotions

| Variables | | Achievements | | | | | | | | | |
| --- | --- | --- | --- | --- | --- | --- | --- | --- | --- | --- | --- |
|  |  | 1 | 2 | 3 | 4 | 5 | 6 | 7 | 8 | 9 | 10 |
| 1. Hand sanitiser placed where necessary | Importance | .445* | .277* | .196* | .325* | .272* | .176* | .303* | .098* | .177* | .199* |
| 2. Regular HH education |  | .280* | .444* | .426* | .421* | .390* | .379* | .394* | .350* | .391* | .387* |
| 3. Practical training by situation |  | .261* | .411* | .407* | .409* | .371* | .375* | .386* | .326* | .391* | .415* |
| 4. Frequent monitoring |  | .229* | .425* | .402* | .404* | .350* | .349* | .353* | .360* | .397* | .406* |
| 5. Department-wide feedback |  | .258* | .402* | .412* | .414* | .411* | .389* | .383* | .345* | .392* | .403* |
| 6. Personal feedback |  | .253* | .374* | .375* | .369* | .360* | .365* | .328* | .326* | .391* | .385* |
| 7. HH information poster |  | .240* | .439* | .462* | .387* | .397* | .438* | .478* | .423* | .441* | .428* |
| 8. Audiovisual alarming/guidance |  | .220* | .422* | .458* | .399* | .411* | .456* | .450* | .490* | .488* | .481* |
| 9. Management’s interest and encouragement |  | .242* | .401* | .410* | .397* | .359* | .370* | .399* | .370* | .473* | .433* |
| 10. Reward and publicise excellent hand hygiene in employees/departments |  | .298* | .390* | .380* | .379* | .390* | .381* | .379* | .342* | .374* | .403* |

* *P* value <0.01

**Supplemental Table A3.** The post hoc analysis results for the relationship between importance and achievement

[Importance]

| Variables | Difference | Differences of means | SE of difference | 95% CI | | *P* value |
| --- | --- | --- | --- | --- | --- | --- |
|  |  |  |  | Lower | Upper |  |
| Hand sanitiser placed where necessary | Nurse - Physician | -0.060 | 0.042 | -0.16 | 0.04 | 0.351 |
|  | Nurse - Other healthcare worker | 0.098 | 0.053 | -0.03 | 0.23 | 0.181 |
|  | Doctor - Other healthcare worker | 0.159 | 0.062 | 0.01 | 0.31 | 0.038 |
| Regular HH education | Nurse - Physician | 0.092 | 0.063 | -0.06 | 0.25 | 0.344 |
|  | Nurse - Other healthcare worker | -0.021 | 0.080 | -0.22 | 0.18 | 0.967 |
|  | Doctor - Other healthcare worker | -0.113 | 0.094 | -0.34 | 0.12 | 0.483 |
| Practical training by situation | Nurse - Physician | 0.155 | 0.066 | -0.01 | 0.32 | 0.066 |
|  | Nurse - Other healthcare worker | 0.061 | 0.085 | -0.15 | 0.27 | 0.773 |
|  | Doctor - Other healthcare worker | -0.094 | 0.099 | -0.34 | 0.15 | 0.636 |
| Frequent monitoring | Nurse - Physician | 0.013 | 0.071 | -0.16 | 0.19 | 0.984 |
|  | Nurse - Other healthcare worker | -0.028 | 0.091 | -0.25 | 0.20 | 0.954 |
|  | Doctor - Other healthcare worker | -0.041 | 0.106 | -0.30 | 0.22 | 0.929 |
| Department-wide feedback | Nurse - Physician | 0.174 | 0.066 | 0.01 | 0.33 | 0.030 |
|  | Nurse - Other healthcare worker | 0.101 | 0.084 | -0.10 | 0.31 | 0.483 |
|  | Doctor - Other healthcare worker | -0.072 | 0.098 | -0.31 | 0.17 | 0.760 |
| Personal feedback | Nurse - Physician | -0.028 | 0.065 | -0.19 | 0.13 | 0.910 |
|  | Nurse - Other healthcare worker | 0.126 | 0.083 | -0.08 | 0.33 | 0.315 |
|  | Doctor - Other healthcare worker | 0.154 | 0.096 | -0.08 | 0.39 | 0.279 |
| HH information poster | Nurse - Physician | 0.299 | 0.072 | 0.12 | 0.48 | 0.000 |
|  | Nurse - Other healthcare worker | -0.079 | 0.093 | -0.31 | 0.15 | 0.693 |
|  | Doctor - Other healthcare worker | -0.378 | 0.108 | -0.64 | -0.11 | 0.002 |
| Audiovisual alarming/guidance | Nurse - Physician | 0.355 | 0.077 | 0.17 | 0.54 | 0.000 |
|  | Nurse - Other healthcare worker | -0.010 | 0.098 | -0.25 | 0.23 | 0.995 |
|  | Doctor - Other healthcare worker | -0.365 | 0.114 | -0.65 | -0.09 | 0.006 |
| Management’s interest and encouragement | Nurse - Physician | 0.271 | 0.077 | 0.08 | 0.46 | 0.002 |
|  | Nurse - Other healthcare worker | -0.047 | 0.098 | -0.29 | 0.19 | 0.892 |
|  | Doctor - Other healthcare worker | -0.317 | 0.114 | -0.60 | -0.04 | 0.021 |
| Reward and publicise excellent hand hygiene in employees/departments | Nurse - Physician | 0.364 | 0.067 | 0.20 | 0.53 | 0.000 |
|  | Nurse - Other healthcare worker | 0.095 | 0.086 | -0.12 | 0.31 | 0.546 |
|  | Doctor - Other healthcare worker | -0.269 | 0.100 | -0.51 | -0.02 | 0.028 |

SE, standard error; CI, confidence interval.

[Achievement]

| Variables | Difference | Differences of means | SE of difference | 95% CI | | *P* value |
| --- | --- | --- | --- | --- | --- | --- |
|  |  |  |  | Lower | Upper |  |
| Hand sanitiser placed where necessary | Nurse - Physician | 0.394 | 0.052 | 0.27 | 0.52 | 0.000 |
|  | Nurse - Other healthcare worker | 0.194 | 0.067 | 0.03 | 0.36 | 0.015 |
|  | Doctor - Other healthcare worker | -0.200 | 0.078 | -0.39 | -0.01 | 0.037 |
| Regular HH education | Nurse - Physician | 0.524 | 0.064 | 0.37 | 0.68 | 0.000 |
|  | Nurse - Other healthcare worker | 0.090 | 0.082 | -0.11 | 0.29 | 0.547 |
|  | Doctor - Other healthcare worker | -0.433 | 0.095 | -0.67 | -0.20 | 0.000 |
| Practical training by situation | Nurse - Physician | 0.668 | 0.072 | 0.49 | 0.85 | 0.000 |
|  | Nurse - Other healthcare worker | 0.109 | 0.093 | -0.12 | 0.34 | 0.508 |
|  | Doctor - Other healthcare worker | -0.560 | 0.108 | -0.82 | -0.29 | 0.000 |
| Frequent monitoring | Nurse - Physician | 0.528 | 0.062 | 0.38 | 0.68 | 0.000 |
|  | Nurse - Other healthcare worker | 0.198 | 0.080 | 0.00 | 0.39 | 0.048 |
|  | Doctor - Other healthcare worker | -0.330 | 0.093 | -0.56 | -0.10 | 0.002 |
| Department-wide feedback | Nurse - Physician | 0.762 | 0.066 | 0.60 | 0.92 | 0.000 |
|  | Nurse - Other healthcare worker | 0.238 | 0.086 | 0.03 | 0.45 | 0.021 |
|  | Doctor - Other healthcare worker | -0.524 | 0.099 | -0.77 | -0.28 | 0.000 |
| Personal feedback | Nurse - Physician | 0.869 | 0.075 | 0.68 | 1.05 | 0.000 |
|  | Nurse - Other healthcare worker | 0.137 | 0.097 | -0.10 | 0.37 | 0.371 |
|  | Doctor - Other healthcare worker | -0.732 | 0.113 | -1.01 | -0.46 | 0.000 |
| HH information poster | Nurse - Physician | 0.545 | 0.065 | 0.39 | 0.70 | 0.000 |
|  | Nurse - Other healthcare worker | 0.069 | 0.084 | -0.14 | 0.27 | 0.713 |
|  | Doctor - Other healthcare worker | -0.476 | 0.097 | -0.71 | -0.24 | 0.000 |
| Audiovisual alarming/guidance | Nurse - Physician | 0.526 | 0.086 | 0.32 | 0.74 | 0.000 |
|  | Nurse - Other healthcare worker | 0.035 | 0.111 | -0.24 | 0.31 | 0.950 |
|  | Doctor - Other healthcare worker | -0.491 | 0.128 | -0.80 | -0.18 | 0.001 |
| Management’s interest and encouragement | Nurse - Physician | 0.598 | 0.080 | 0.40 | 0.79 | 0.000 |
|  | Nurse - Other healthcare worker | 0.026 | 0.103 | -0.23 | 0.28 | 0.968 |
|  | Doctor - Other healthcare worker | -0.572 | 0.119 | -0.86 | -0.28 | 0.000 |
| Reward and publicise excellent hand hygiene employees/departments | Nurse - Physician | 0.447 | 0.076 | 0.26 | 0.63 | 0.000 |
|  | Nurse - Other healthcare worker | -0.007 | 0.097 | -0.25 | 0.23 | 0.997 |
|  | Doctor - Other healthcare worker | -0.454 | 0.113 | -0.73 | -0.18 | 0.000 |

SE, standard error; CI, confidence interval.

**Supplemental Table A4.** The post hoc analysis results for knowledge, attitudes, and behaviours about hand hygiene among healthcare workers

| Variables | Difference | Differences of means | SE of difference | 95% CI | | *P* value |
| --- | --- | --- | --- | --- | --- | --- |
|  |  |  |  | Lower | Upper |  |
| Hand hygiene is important to maintaining my professionalism. | Nurse - Physician | 0.126 | 0.080 | -0.07 | 0.32 | 0.288 |
|  | Nurse - Other healthcare worker | 0.362 | 0.103 | 0.11 | 0.62 | 0.002 |
|  | Doctor - Other healthcare worker | 0.236 | 0.120 | -0.06 | 0.53 | 0.145 |
| I know when to do hand hygiene. | Nurse - Physician | 0.234 | 0.068 | 0.07 | 0.40 | 0.003 |
|  | Nurse - Other healthcare worker | 0.308 | 0.088 | 0.09 | 0.52 | 0.002 |
|  | Doctor - Other healthcare worker | 0.074 | 0.102 | -0.18 | 0.32 | 0.770 |
| I know the correct hand hygiene method (action). | Nurse - Physician | 0.264 | 0.068 | 0.10 | 0.43 | 0.001 |
|  | Nurse - Other healthcare worker | 0.291 | 0.088 | 0.07 | 0.51 | 0.005 |
|  | Doctor - Other healthcare worker | 0.027 | 0.102 | -0.22 | 0.28 | 0.965 |
| I do hand hygiene before contacting the patient. | Nurse - Physician | 0.455 | 0.083 | 0.25 | 0.66 | 0.000 |
|  | Nurse - Other healthcare worker | 0.355 | 0.107 | 0.09 | 0.62 | 0.004 |
|  | Doctor - Other healthcare worker | -0.1 | 0.124 | -0.41 | 0.20 | 0.722 |
| Hand hygiene is a part of medical practice. | Nurse - Physician | 0.145 | 0.073 | -0.03 | 0.32 | 0.143 |
|  | Nurse - Other healthcare worker | 0.211 | 0.095 | -0.02 | 0.44 | 0.084 |
|  | Doctor - Other healthcare worker | 0.066 | 0.11 | -0.20 | 0.34 | 0.833 |
| It is easy to cleanse hands because alcohol gel is close. | Nurse - Physician | 0.447 | 0.079 | 0.25 | 0.64 | 0.000 |
|  | Nurse - Other healthcare worker | 0.392 | 0.102 | 0.14 | 0.64 | 0.001 |
|  | Doctor - Other healthcare worker | -0.055 | 0.118 | -0.34 | 0.23 | 0.896 |
| If I do not do hand hygiene, I can get infected too. | Nurse - Physician | 0.202 | 0.072 | 0.03 | 0.38 | 0.020 |
|  | Nurse - Other healthcare worker | 0.137 | 0.093 | -0.09 | 0.37 | 0.343 |
|  | Doctor - Other healthcare worker | -0.066 | 0.108 | -0.33 | 0.20 | 0.832 |
| My patient expects me to do my hand hygiene well. | Nurse - Physician | 0.205 | 0.08 | 0.01 | 0.40 | 0.038 |
|  | Nurse - Other healthcare worker | 0.123 | 0.104 | -0.13 | 0.38 | 0.495 |
|  | Doctor - Other healthcare worker | -0.082 | 0.12 | -0.38 | 0.21 | 0.790 |
| I believe that hand hygiene blocks the spread of infection. | Nurse - Physician | 0.148 | 0.073 | -0.03 | 0.33 | 0.130 |
|  | Nurse - Other healthcare worker | 0.168 | 0.095 | -0.06 | 0.40 | 0.206 |
|  | Doctor - Other healthcare worker | 0.020 | 0.11 | -0.25 | 0.29 | 0.984 |
| The performance of hand hygiene by colleagues affects my performance. | Nurse - Physician | 0.458 | 0.096 | 0.22 | 0.69 | 0.000 |
|  | Nurse - Other healthcare worker | 0.196 | 0.124 | -0.11 | 0.50 | 0.288 |
|  | Doctor - Other healthcare worker | -0.262 | 0.144 | -0.61 | 0.09 | 0.191 |
| Jewellery and artificial nails make your hands more germ-friendly. | Nurse - Physician | 0.056 | 0.083 | -0.15 | 0.26 | 0.798 |
|  | Nurse - Other healthcare worker | 0.221 | 0.107 | -0.04 | 0.48 | 0.118 |
|  | Doctor - Other healthcare worker | 0.166 | 0.124 | -0.14 | 0.47 | 0.408 |

SE, standard error; CI, confidence interval.

**Supplemental Table A5.** The post hoc analysis results for internal and emotional motivation regarding hand hygiene among healthcare workers

| Variables | Difference | Differences of means | SE of difference | 95% CI | | *P* value |
| --- | --- | --- | --- | --- | --- | --- |
|  |  |  |  | Lower | Upper |  |
| I do hand hygiene to become a role model to my colleagues. | Nurse - Physician | 0.509 | 0.134 | 0.18 | 0.84 | 0.001 |
|  | Nurse - Other healthcare worker | 0.622 | 0.174 | 0.20 | 1.05 | 0.002 |
|  | Doctor - Other healthcare worker | 0.113 | 0.201 | -0.38 | 0.61 | 0.853 |
| Hand hygiene posters and screensavers help with hand hygiene. | Nurse - Physician | 0.583 | 0.115 | 0.30 | 0.86 | 0.000 |
|  | Nurse - Other healthcare worker | -0.042 | 0.148 | -0.41 | 0.32 | 0.961 |
|  | Doctor - Other healthcare worker | -0.625 | 0.172 | -1.05 | -0.20 | 0.001 |
| I want to receive feedback on hand hygiene and improve my performance. | Nurse - Physician | 0.168 | 0.112 | -0.11 | 0.44 | 0.327 |
|  | Nurse - Other healthcare worker | 0.183 | 0.145 | -0.17 | 0.54 | 0.454 |
|  | Doctor - Other healthcare worker | 0.015 | 0.168 | -0.40 | 0.43 | 0.996 |
| I can do better hand hygiene if the sink is near. | Nurse - Physician | 0.459 | 0.109 | 0.19 | 0.73 | 0.000 |
|  | Nurse - Other healthcare worker | 0.077 | 0.141 | -0.27 | 0.42 | 0.862 |
|  | Doctor - Other healthcare worker | -0.382 | 0.163 | -0.78 | 0.02 | 0.065 |
| Soap or hand towels are provided in each hospital room for good hand hygiene. | Nurse - Physician | 0.433 | 0.134 | 0.10 | 0.76 | 0.006 |
|  | Nurse - Other healthcare worker | -0.099 | 0.173 | -0.52 | 0.33 | 0.848 |
|  | Doctor - Other healthcare worker | -0.533 | 0.201 | -1.02 | -0.04 | 0.030 |
| If I could get a promotion for hand hygiene, I would do better hand hygiene. | Nurse - Physician | 0.486 | 0.129 | 0.17 | 0.80 | 0.001 |
|  | Nurse - Other healthcare worker | 0.457 | 0.167 | 0.05 | 0.87 | 0.024 |
|  | Doctor - Other healthcare worker | -0.029 | 0.193 | -0.50 | 0.44 | 0.989 |
| Our hospital staff regularly receive feedback on hand hygiene practices. | Nurse - Physician | 0.929 | 0.099 | 0.69 | 1.17 | 0.000 |
|  | Nurse - Other healthcare worker | 0.261 | 0.127 | -0.05 | 0.57 | 0.123 |
|  | Doctor - Other healthcare worker | -0.668 | 0.147 | -1.03 | -0.31 | 0.000 |
| Hand hygiene is the most important in my work. | Nurse - Physician | 1.074 | 0.105 | 0.82 | 1.33 | 0.000 |
|  | Nurse - Other healthcare worker | 0.172 | 0.136 | -0.16 | 0.51 | 0.451 |
|  | Doctor - Other healthcare worker | -0.902 | 0.158 | -1.29 | -0.51 | 0.000 |

SE, standard error; CI, confidence interval.

**Supplemental Table A6.** The post hoc analysis results for the barriers to hand hygiene compliance

| Variables | Difference | Differences of means | SE of difference | 95% CI | | *P* value |
| --- | --- | --- | --- | --- | --- | --- |
|  |  |  |  | Lower | Upper |  |
| Hand hygiene makes your hands painful and dry (skin trouble) | Nurse - Physician | 0.78 | 0.145 | 0.42 | 1.14 | 0.000 |
|  | Nurse - Other healthcare worker | 0.336 | 0.188 | -0.13 | 0.80 | 0.205 |
|  | Doctor - Other healthcare worker | -0.444 | 0.218 | -0.98 | 0.09 | 0.125 |
| It is difficult to do hand hygiene if a superior does not do hand hygiene | Nurse - Physician | -0.329 | 0.143 | -0.68 | 0.02 | 0.072 |
|  | Nurse - Other healthcare worker | 0.045 | 0.186 | -0.41 | 0.50 | 0.971 |
|  | Doctor - Other healthcare worker | 0.375 | 0.215 | -0.15 | 0.90 | 0.219 |
| Hand hygiene is difficult in an emergency | Nurse - Physician | 0.413 | 0.131 | 0.09 | 0.74 | 0.007 |
|  | Nurse - Other healthcare worker | 0.951 | 0.170 | 0.53 | 1.37 | 0.000 |
|  | Doctor - Other healthcare worker | 0.538 | 0.197 | 0.06 | 1.02 | 0.024 |
| It is hard to tell my colleagues to do hand hygiene | Nurse - Physician | -0.142 | 0.140 | -0.49 | 0.20 | 0.600 |
|  | Nurse - Other healthcare worker | 0.211 | 0.182 | -0.23 | 0.66 | 0.509 |
|  | Doctor - Other healthcare worker | 0.353 | 0.21 | -0.16 | 0.87 | 0.244 |
| Hand hygiene wastes time for more important things. | Nurse - Physician | 0.442 | 0.137 | 0.11 | 0.78 | 0.006 |
|  | Nurse - Other healthcare worker | 0.701 | 0.177 | 0.27 | 1.14 | 0.000 |
|  | Doctor - Other healthcare worker | 0.260 | 0.205 | -0.24 | 0.76 | 0.447 |
| Hand hygiene is not necessary if you wear gloves | Nurse - Physician | -0.535 | 0.129 | -0.85 | -0.22 | 0.000 |
|  | Nurse - Other healthcare worker | 0.003 | 0.167 | -0.41 | 0.41 | 1.000 |
|  | Doctor - Other healthcare worker | 0.538 | 0.193 | 0.06 | 1.01 | 0.021 |
| I don't think there's any ethical problem even if I don't sterilise my hands | Nurse - Physician | -0.070 | 0.125 | -0.38 | 0.24 | 0.855 |
|  | Nurse - Other healthcare worker | 0.049 | 0.161 | -0.35 | 0.44 | 0.955 |
|  | Doctor - Other healthcare worker | 0.119 | 0.187 | -0.34 | 0.58 | 0.816 |
| Hand hygiene has not become a habit. | Nurse - Physician | -0.486 | 0.132 | -0.81 | -0.16 | 0.001 |
|  | Nurse - Other healthcare worker | -0.196 | 0.171 | -0.61 | 0.22 | 0.516 |
|  | Doctor - Other healthcare worker | 0.29 | 0.197 | -0.19 | 0.77 | 0.340 |
| I often forget about hand hygiene. | Nurse - Physician | -0.610 | 0.132 | -0.93 | -0.29 | 0.000 |
|  | Nurse - Other healthcare worker | -0.009 | 0.171 | -0.43 | 0.41 | 0.999 |
|  | Doctor - Other healthcare worker | 0.602 | 0.198 | 0.12 | 1.09 | 0.010 |
| There is no special disadvantage even if hand hygiene is not done | Nurse - Physician | -0.541 | 0.13 | -0.86 | -0.22 | 0.000 |
|  | Nurse - Other healthcare worker | 0.007 | 0.169 | -0.41 | 0.42 | 0.999 |
|  | Doctor - Other healthcare worker | 0.547 | 0.195 | 0.07 | 1.03 | 0.020 |
| I don't know exactly when to do hand hygiene. | Nurse - Physician | -0.387 | 0.120 | -0.68 | -0.09 | 0.005 |
|  | Nurse - Other healthcare worker | -0.008 | 0.155 | -0.39 | 0.37 | 0.999 |
|  | Doctor - Other healthcare worker | 0.379 | 0.179 | -0.06 | 0.82 | 0.107 |
| If hand hygiene is being monitored, I don't want to do hand hygiene. | Nurse - Physician | 0.061 | 0.156 | -0.32 | 0.44 | 0.925 |
|  | Nurse - Other healthcare worker | 0.337 | 0.201 | -0.16 | 0.83 | 0.248 |
|  | Doctor - Other healthcare worker | 0.275 | 0.233 | -0.30 | 0.85 | 0.498 |
| I'm not sure if hand hygiene is helpful for patient safety | Nurse - Physician | -0.101 | 0.122 | -0.40 | 0.20 | 0.712 |
|  | Nurse - Other healthcare worker | 0.085 | 0.158 | -0.30 | 0.47 | 0.865 |
|  | Doctor - Other healthcare worker | 0.186 | 0.183 | -0.26 | 0.63 | 0.596 |
| Because there is no soap or hand towel in each hospital room, proper hand hygiene is difficult. | Nurse - Physician | -0.551 | 0.144 | -0.9 | -0.20 | 0.001 |
|  | Nurse - Other healthcare worker | 0.058 | 0.186 | -0.4 | 0.51 | 0.952 |
|  | Doctor - Other healthcare worker | 0.609 | 0.215 | 0.08 | 1.14 | 0.018 |
| Hand hygiene makes your hands painful and dry (skin trouble) | Nurse - Physician | 0.780 | 0.145 | 0.42 | 1.14 | 0.000 |
|  | Nurse - Other healthcare worker | 0.336 | 0.188 | -0.13 | 0.80 | 0.205 |
|  | Doctor - Other healthcare worker | -0.444 | 0.218 | -0.98 | 0.09 | 0.125 |

SE, standard error; CI, confidence interval.

**Supplemental Table A7.** Measures for overcoming barriers to performing hand hygiene (all ages)

| Method | Total | Nurse | Physician | Other healthcare workers |
| --- | --- | --- | --- | --- |
| Diversify types of hand sanitisers. | 196 (19.9) | 126 (18.3) | 51 (26.3) | 19 (18.6) |
| Remind timing of hand hygiene through a reminder. | 96 (9.8) | 53 (7.7) | 31 (16.0) | 12 (11.8) |
| Induce an atmosphere of requesting hand hygiene from staff through patient and caregiver education. | 76 (7.7) | 59 (8.6) | 11 (5.7) | 6 (5.9) |
| Change perception through various hand hygiene campaigns. | 178 (18.1) | 130 (18.9) | 24 (12.4) | 24 (23.5) |
| Hand hygiene results are reflected in the personal review. | 26 (2.6) | 18 (2.6) | 4 (2.1) | 4 (3.9) |
| Provide on-the-spot feedback on the observation. | 79 (8.0) | 58 (8.4) | 18 (9.3) | 3 (2.9) |
| Monitoring is carried out on a regular basis. | 44 (4.5) | 25(3.6) | 14(7.2) | 5 (4.9) |
| Install soap and paper towels in each hospital room. | 180 (18.3) | 139 (20.2) | 25 (12.9) | 16 (15.7) |
| Hand hygiene real-name system to manage personal performance rate. | 26 (2.6) | 18 (2.6) | 6 (3.1) | 2 (2.0) |
| Conduct a peer-to-peer assessment of performance rates. | 12 (1.2) | 11 (1.6) | 1 (0.5) | 0 (0.0) |
| Strengthen hand hygiene theory education. | 13 (1.3) | 10 (1.5) | 2 (1.0) | 1 (1.0) |
| Practical training for strengthening hand hygiene compliance. | 57 (5.8) | 40 (5.8) | 7 (3.6) | 10 (9.8) |

Data are presented as numbers (%) of participants.

**Supplemental Table A8.** Measures for overcoming barriers to performing hand hygiene (twenties)

| Method | Total | Nurse | Physician | Other healthcare workers |
| --- | --- | --- | --- | --- |
| Diversify types of hand sanitisers. | 86 (21.7) | 65 (20.6) | 15 (27.3) | 6 (23.1) |
| Remind timing of hand hygiene through a reminder. | 31 (7.8) | 15 (4.7) | 12 (21.8) | 4 (15.4) |
| Induce an atmosphere of requesting hand hygiene from staff through patient and caregiver education. | 29 (7.3) | 23 (7.3) | 5 (9.1) | 1 (3.8) |
| Change perception through various hand hygiene campaigns. | 63 (15.9) | 55 (17.4) | 7 (12.7) | 1 (3.8) |
| Hand hygiene results are reflected in the personal review. | 9 (2.3) | 7 (2.2) | 1 (1.8) | 1 (3.8) |
| Provide on-the-spot feedback on the observation. | 27 (6.8) | 25 (7.9) | 1 (1.8) | 1 (3.8) |
| Monitoring is carried out on a regular basis. | 15 (3.8) | 10 (3.2) | 2 (3.6) | 3 (11.5) |
| Install soap and paper towels in each hospital room. | 101 (25.4) | 86 (27.2) | 9 (16.4) | 6 (23.1) |
| Hand hygiene real-name system to manage personal performance rate. | 8 (2) | 7 (2.2) | 0 (0) | 1 (3.8) |
| Conduct a peer-to-peer assessment of performance rates. | 6 (1.5) | 5 (1.6) | 1 (1.8) | 0 (0) |
| Strengthen hand hygiene theory education. | 3 (0.8) | 3 (0.9) | 0 (0) | 0 (0) |
| Practical training for strengthening hand hygiene compliance. | 19 (4.8) | 15 (4.7) | 2 (3.6) | 2 (7.7) |

Data are presented as numbers (%) of participants.

**Supplemental Table A9.** Measures for overcoming barriers to performing hand hygiene (30s)

| Method | Total | Nurse | Physician | Other healthcare workers |
| --- | --- | --- | --- | --- |
| Diversify types of hand sanitisers. | 60 (20.6) | 38 (20.3) | 17 (23.3) | 5 (16.1) |
| Remind timing of hand hygiene through a reminder. | 25 (8.6) | 13 (7) | 8 (11) | 4 (12.9) |
| Induce an atmosphere of requesting hand hygiene from staff through patient and caregiver education. | 25 (8.6) | 19 (10.2) | 4 (5.5) | 2 (6.5) |
| Change perception through various hand hygiene campaigns. | 47 (16.2) | 32 (17.1) | 8 (11) | 7 (22.6) |
| Hand hygiene results are reflected in the personal review. | 9 (3.1) | 6 (3.2) | 2 (2.7) | 1 (3.2) |
| Provide on-the-spot feedback on the observation. | 21 (7.2) | 10 (5.3) | 9 (12.3) | 2 (6.5) |
| Monitoring is carried out on a regular basis. | 14 (4.8) | 7 (3.7) | 5 (6.8) | 2 (6.5) |
| Install soap and paper towels in each hospital room. | 55 (18.9) | 39 (20.9) | 11 (15.1) | 5 (16.1) |
| Hand hygiene real-name system to manage personal performance rate. | 10 (3.4) | 4 (2.1) | 5 (6.8) | 1 (3.2) |
| Conduct a peer-to-peer assessment of performance rates. | 3 (1) | 3 (1.6) | 0 (0) | 0 (0) |
| Strengthen hand hygiene theory education. | 5 (1.7) | 3 (1.6) | 1 (1.4) | 1 (3.2) |
| Practical training for strengthening hand hygiene compliance. | 17 (5.8) | 13 (7) | 3 (4.1) | 1 (3.2) |

Data are presented as numbers (%) of participants.

**Supplemental Table A10.** Measures for overcoming barriers to performing hand hygiene (forties)

| Method | Total | Nurse | Physician | Other healthcare workers |
| --- | --- | --- | --- | --- |
| Diversify types of hand sanitisers. | 41 (18.4) | 22 (14.9) | 14 (29.8) | 5 (17.9) |
| Remind timing of hand hygiene through a reminder. | 32 (14.3) | 22 (14.9) | 9 (19.1) | 1 (3.6) |
| Induce an atmosphere of requesting hand hygiene from staff through patient and caregiver education. | 21 (9.4) | 17 (11.5) | 2 (4.3) | 2 (7.1) |
| Change perception through various hand hygiene campaigns. | 51 (22.9) | 36 (24.3) | 3 (6.4) | 12 (42.9) |
| Hand hygiene results are reflected in the personal review. | 6 (2.7) | 4 (2.7) | 1 (2.1) | 1 (3.6) |
| Provide on-the-spot feedback on the observation. | 22 (9.9) | 15 (10.1) | 7 (14.9) | 0 (0) |
| Monitoring is carried out on a regular basis. | 7 (3.1) | 3 (2) | 4 (8.5) | 0 (0) |
| Install soap and paper towels in each hospital room. | 18 (8.1) | 11 (7.4) | 5 (10.6) | 2 (7.1) |
| Hand hygiene real-name system to manage personal performance rate. | 6 (2.7) | 5 (3.4) | 1 (2.1) | 0 (0) |
| Conduct a peer-to-peer assessment of performance rates. | 2 (0.9) | 2 (1.4) | 0 (0) | 0 (0) |
| Strengthen hand hygiene theory education. | 5 (2.2) | 4 (2.7) | 1 (2.1) | 0 (0) |
| Practical training for strengthening hand hygiene compliance. | 12 (5.4) | 7 (4.7) | 0 (0) | 5 (17.9) |

Data are presented as numbers (%) of participants.

**Supplemental Table A11.** Measures for overcoming barriers to performing hand hygiene (fifties)

| Method | Total | Nurse | Physician | Other healthcare workers |
| --- | --- | --- | --- | --- |
| Diversify types of hand sanitisers. | 6 (9) | 1 (2.9) | 2 (12.5) | 3 (17.6) |
| Remind timing of hand hygiene through a reminder. | 7 (10.4) | 2 (5.9) | 2 (12.5) | 3 (17.6) |
| Induce an atmosphere of requesting hand hygiene from staff through patient and caregiver education. | 1 (1.5) | 0 (0) | 0 (0) | 1 (5.9) |
| Change perception through various hand hygiene campaigns. | 16 (23.9) | 6 (17.6) | 6 (37.5) | 4 (23.5) |
| Hand hygiene results are reflected in the personal review. | 2 (3) | 1 (2.9) | 0 (0) | 1 (5.9) |
| Provide on-the-spot feedback on the observation. | 9 (13.4) | 8 (23.5) | 1 (6.3) | 0 (0) |
| Monitoring is carried out on a regular basis. | 8 (11.9) | 5 (14.7) | 3 (18.8) | 0 (0) |
| Install soap and paper towels in each hospital room. | 6 (9) | 3 (8.8) | 0 (0) | 3 (17.6) |
| Hand hygiene real-name system to manage personal performance rate. | 2 (3) | 2 (5.9) | 0 (0) | 0 (0) |
| Conduct a peer-to-peer assessment of performance rates. | 1 (1.5) | 1 (2.9) | 0 (0) | 0 (0) |
| Strengthen hand hygiene theory education. | 0 (0) | 0 (0) | 0 (0) | 0 (0) |
| Practical training for strengthening hand hygiene compliance. | 9 (13.4) | 5 (14.7) | 2 (12.5) | 2 (11.8) |

Data are presented as numbers (%) of participants.

**Supplemental Table A12.** The need for external reminders

| Variables | Total | | Nurse | | Physician | | Other healthcare workers | | *P* value |
| --- | --- | --- | --- | --- | --- | --- | --- | --- | --- |
|  | Mean | SD | Mean | SD | Mean | SD | Mean | SD |  |
| I sometimes forget about hand hygiene. | 3.32 | 1.57 | 3.22^b^ | 1.56 | 3.77^a^ | 1.54 | 3.24^b^ | 1.50 | <0.001 |
| The compliance rate will increase if the hospital's administrators, professors, and team leaders do a good job of hand hygiene. | 4.95 | 1.58 | 4.96^a^ | 1.63 | 4.86^a^ | 1.45 | 5.00^a^ | 1.46 | 0.675 |
| If someone is monitoring hand hygiene, compliance will go up. | 4.49 | 1.64 | 4.40^b^ | 1.69 | 4.78^a^ | 1.40 | 4.61^b^ | 1.59 | 0.010 |
| If your colleagues tell you to do hand hygiene, your compliance will increase. | 4.73 | 1.52 | 4.70^a^ | 1.55 | 4.88^a^ | 1.37 | 4.72^a^ | 1.57 | 0.329 |

SD, standard deviation.

^a, b^ indicate a significant mean difference between groups a and b.

**Supplemental Table A13.** The post hoc analysis results regarding the need for external reminders

| Variables | Difference | Differences of means | SE of difference | 95% CI | | *P* value |
| --- | --- | --- | --- | --- | --- | --- |
|  |  |  |  | Lower | Upper |  |
| I do hand hygiene to become a role model to my colleagues. | Nurse - Physician | -0.552 | 0.123 | 0.00 | -0.85 | -0.25 |
|  | Nurse - Other healthcare worker | -0.023 | 0.159 | 0.99 | -0.41 | 0.367 |
|  | Doctor - Other healthcare worker | 0.529 | 0.184 | 0.02 | 0.08 | 0.981 |
| Hand hygiene posters and screensavers help with hand hygiene. | Nurse - Physician | 0.101 | 0.126 | 0.72 | -0.21 | 0.409 |
|  | Nurse - Other healthcare worker | -0.038 | 0.162 | 0.97 | -0.44 | 0.359 |
|  | Doctor - Other healthcare worker | -0.139 | 0.188 | 0.76 | -0.60 | 0.321 |
| I want to receive feedback on hand hygiene and improve my performance. | Nurse - Physician | -0.383 | 0.13 | 0.01 | -0.70 | -0.065 |
|  | Nurse - Other healthcare worker | -0.206 | 0.167 | 0.47 | -0.62 | 0.204 |
|  | Doctor - Other healthcare worker | 0.177 | 0.194 | 0.66 | -0.30 | 0.652 |
| I can do better hand hygiene if the sink is near. | Nurse - Physician | -0.18 | 0.121 | 0.33 | -0.48 | 0.116 |
|  | Nurse - Other healthcare worker | -0.029 | 0.156 | 0.98 | -0.41 | 0.354 |
|  | Doctor - Other healthcare worker | 0.151 | 0.181 | 0.70 | -0.29 | 0.594 |

SE, standard error; CI, confidence interval.
