# Supplementary figures and images for: Hand hygiene knowledge, attitude, barriers and improvement measures among healthcare workers in the Republic of Korea: a cross-sectional survey exploring interprofessional differences
Source: Antimicrob Resist Infect Control. 2023 Sep 7;12:93. doi: 10.1186/s13756-023-01296-y (PMC10483734; doi:10.1186/s13756-023-01296-y)

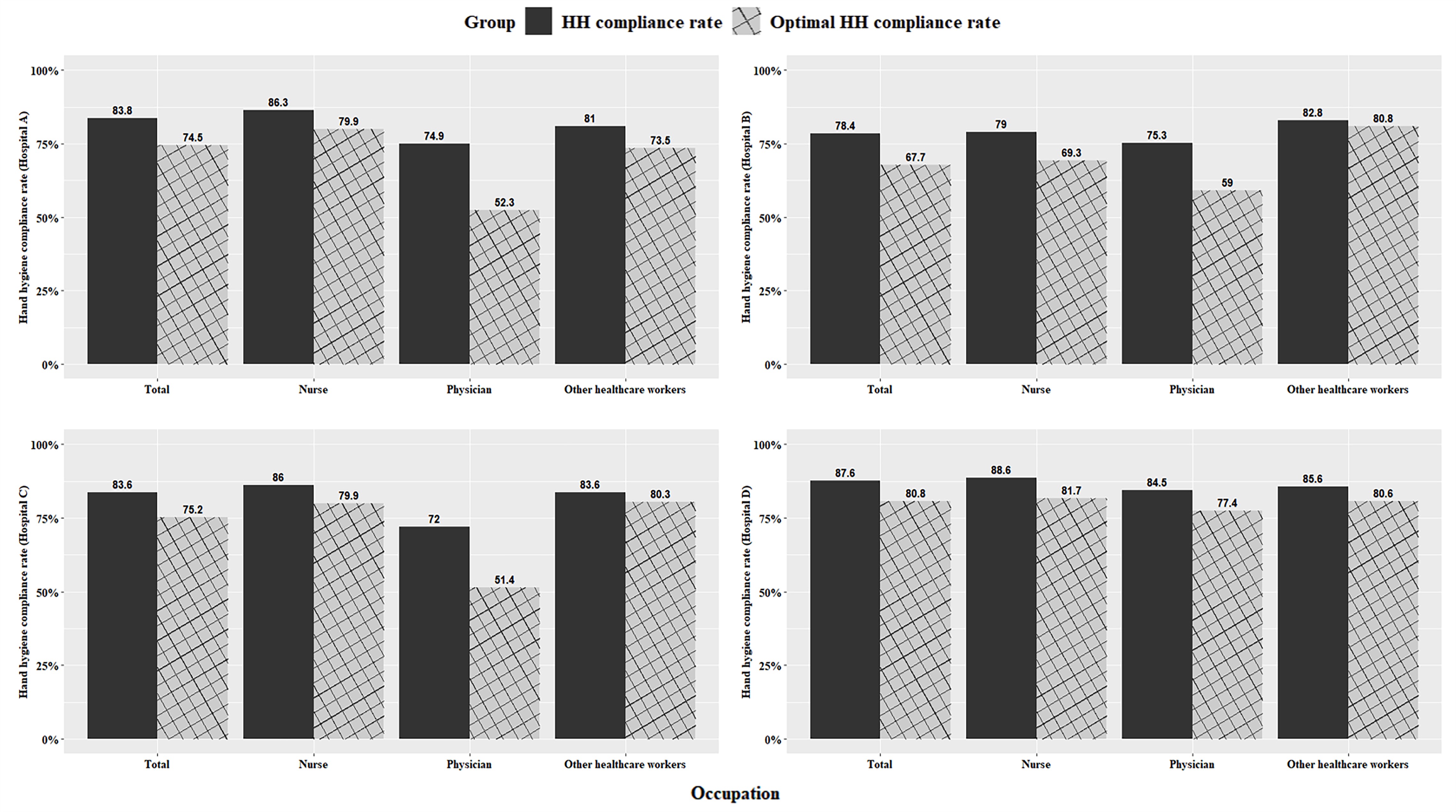

Supplement: Supplementary file 2 — Supplemental Fig. 2. Comparison of education frequency among healthcare workers. [file 13756_2023_1296_MOESM2_ESM.jpg]

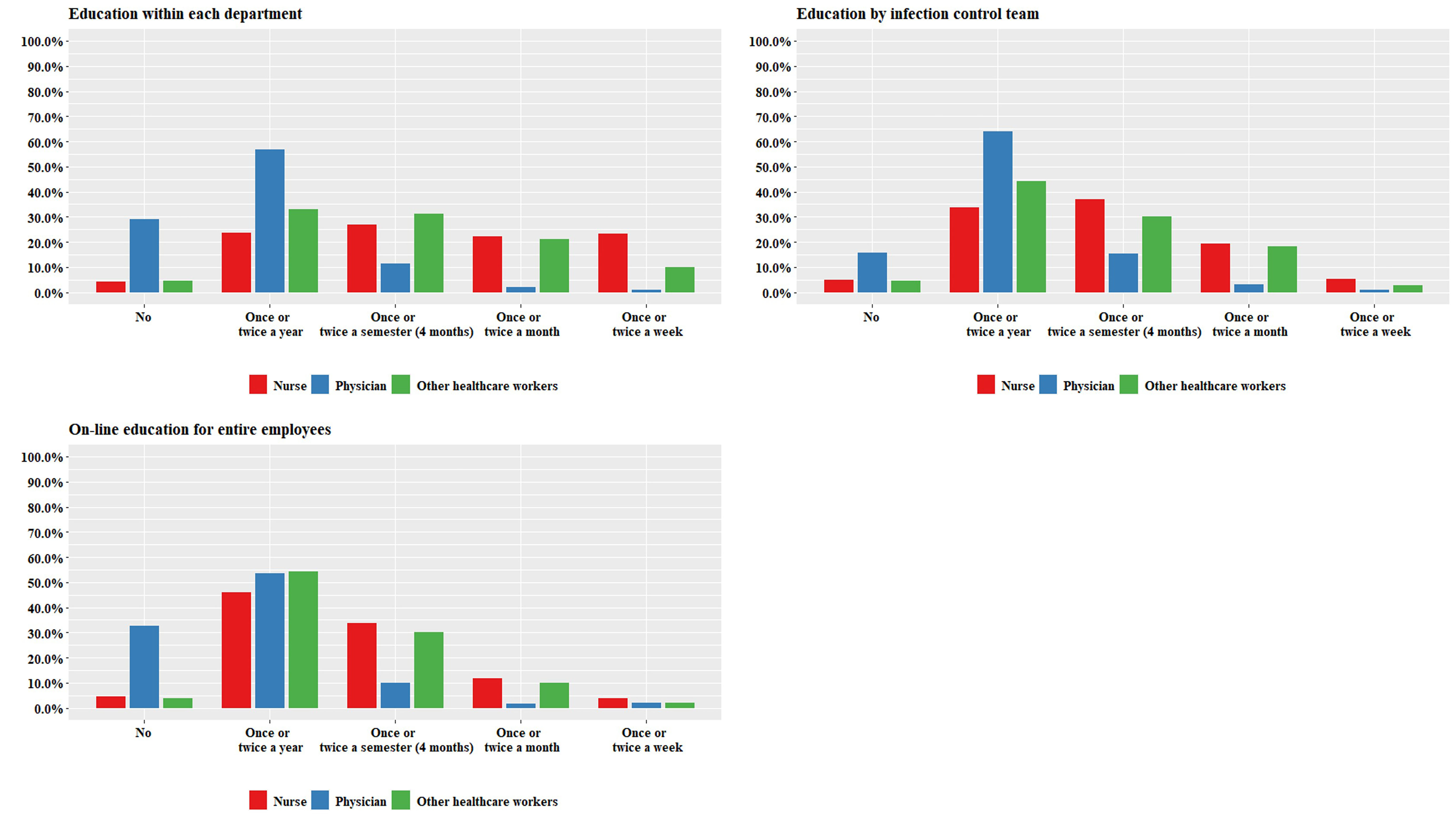

Supplement: Supplementary file 3 — Supplemental Fig. 3. Improvement measures for barriers to performing hand hygiene based on first choice by respondents. The graph shows the percentage of respondents who selected each improvement measure as their first choice. The measures include offering different types of hand sanitisers (1), sending reminders about hand hygiene timing (2), educating patients and caregivers to promote a culture of hand hygiene among staff (3), using hand hygiene campaigns to change perceptions (4), including hand hygiene results in staff performance reviews (5), providing immediate feedback on hand hygiene observations (6), conducting regular monitoring of hand hygiene practices (7), ensuring soap and paper towels are available in all hospital rooms (8), implementing a real-name system to track hand hygiene performance (9), conducting peer-to-peer assessments of hand hygiene performance (10), strengthening hand hygiene theory education (11), and providing training for different hand hygiene situations (12) [file 13756_2023_1296_MOESM3_ESM.jpg]

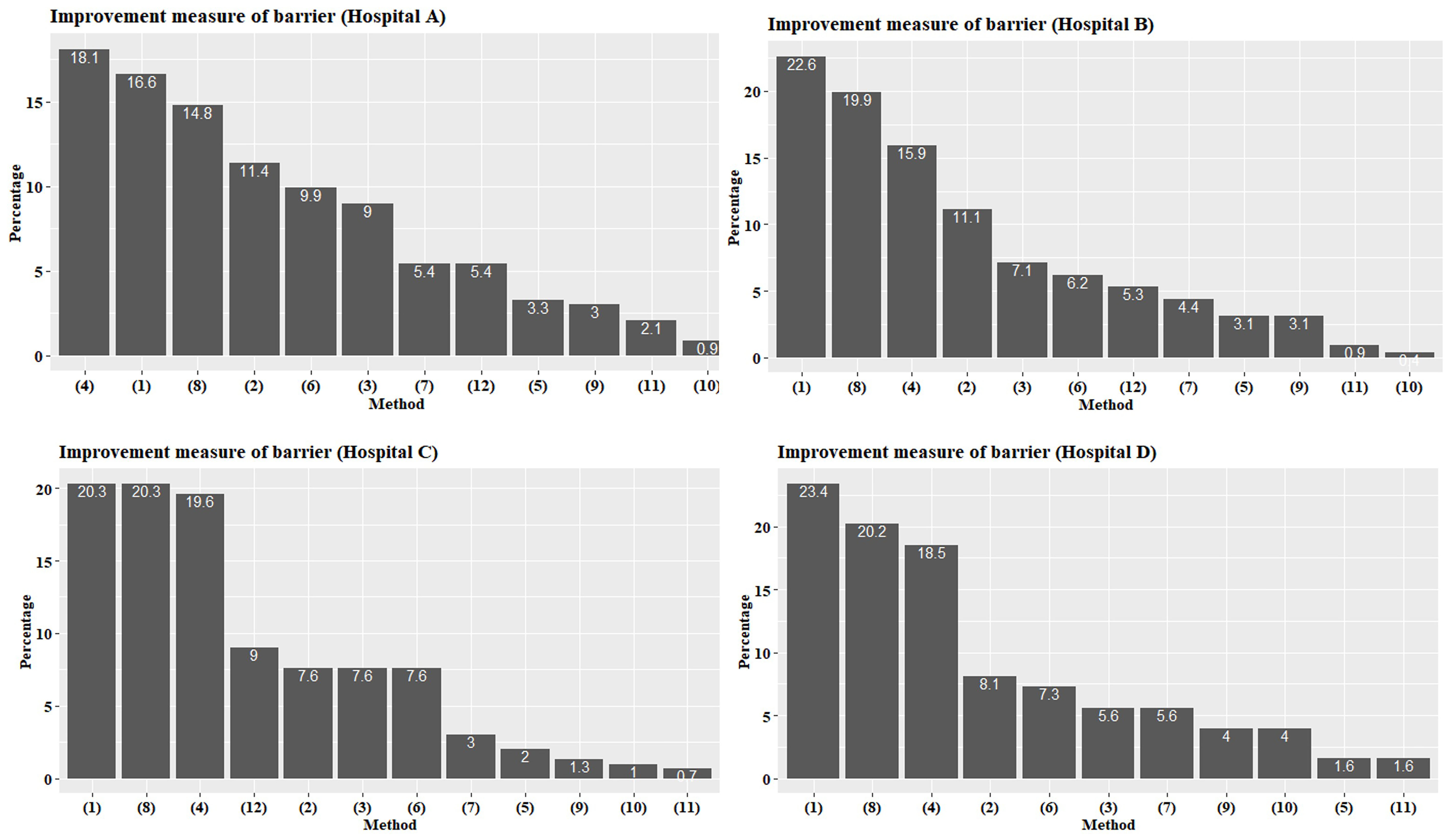

Supplement: Supplementary file 4 — Supplementary Material 4 [file 13756_2023_1296_MOESM4_ESM.jpg]
